# The Specter of Corporate Necromancy: Who Controls the Dead in the Age of Digital Doppelgängers?

**DOI:** 10.1080/15265161.2024.2441755

**Published:** 2025-01-29

**Authors:** Hazem Zohny

**Affiliations:** University of Oxford

The development of digital doppelgängers (DDs)—AI systems trained to replicate individual personalities—raises questions about corporate control over digital representations of the deceased. As language models become better at mimicking human interaction patterns, companies are developing platforms that aim to preserve and commercialize digital personas after death.^1^

In their recent, thought-provoking paper, Iglesias and colleagues argue that DDs could provide a form of persistence that can maintain certain aspects of relationships and legacy (Iglesias et al. 2025). However, they overlook a fundamental risk: the ongoing vulnerability of these digital selves to exploitation, and how this may fundamentally alter our intuitions in some of the thought experiments they pose. This essay will explore how the creation of DDs opens the door to a range of indefinite potential abuses which may undermine the value they might otherwise offer.

## CORPORATE NECROMANCY IN THEORY AND PRACTICE

I use the term “corporate necromancy” to describe the commercial control and exploitation of digital representations of the deceased. While DDs could be misused by various actors—from family members to political groups and religious organizations—corporations warrant particular scrutiny given their existing control over the technical infrastructure and their tendency toward market consolidation in digital technologies.

Firstly, it is worth emphasizing how quickly this technology is advancing, even beyond what Iglesias and colleagues suggest. Two recent studies underscore the striking ease and accuracy of creating realistic DDs. The first, by Park et al. developed “generative agents” that simulate the attitudes and behaviors of 1,052 real individuals by combining AI-conducted two-hour interviews with a large language model (Park et al. 2024). The agents (basically DDs without a cloned voice or avatar) demonstrated a level of consistency in replicating participants’ responses on a range of questionnaires that closely mirrored how consistent the people they represent were with their own answers over time. Relatedly, Pataranutaporn et al.’s “Future You” system used personal data and GPT-3.5 to generate interactive future selves (Pataranutaporn et al. 2024). Participants reported a profound sense of connection to these digital counterparts, with reported reduced anxiety and increased sense of future self-continuity. These findings are strong indicators that creating DDs will not require much technical expertise or effort, especially as language models themselves can be used to conduct interviews (perhaps over days, weeks, months or a lifetime) to gather biographical data.

So how might corporate interests skew intuitions about the attractiveness of DDs as a form of personhood extension? For starters, DD creation at least opens the door to corporations monetizing them in various ways, like offering subscription-based services for grieving families,^2^ using the digital replica’s “voice” for targeted advertising to their family, or having them otherwise endorse products or services. This raises concerns about posthumous autonomy—simply by hosting a DD, a corporation or others using its platform could manipulate a DD to promote ideas or products that the original person would have opposed.

The appropriate regulations could reduce the chances of this happening, but it is worth noting that we already see precedent for such practices in how corporations handle deceased celebrities’ digital rights. Consider, for instance, how Fred Astaire’s image was used to vacuum dance in a Dirt Devil commercial (Staff 1999), or how James Dean was digitally cast in a new film decades after his death (Coyle 2019). DDs would extend this commercial control beyond celebrities to potentially anyone using the technology. A DD could be intentionally reprogrammed to support causes, political stances, or ideologies that are fundamentally opposed to the beliefs held by the original person. This kind of distortion is not just theoretical. The technology, as it stands, could easily be wielded to subtly—or drastically—alter the legacy someone leaves behind through prompt injection.^3^ Opening oneself up to such possibilities by creating a DD is an immense vulnerability, and there’s no obvious mechanism for the deceased to prevent abuse potential.

Technical solutions like encryption or blockchain-based ownership rights cannot fully mitigate this vulnerability, nor can simply keeping them offline. This is because the interactive nature of DDs means they can be replicated through sustained interaction—an adversary could systematically interview a digital doppelgänger, record its responses, and use this data to train a new model that closely mimics the original (although, this is true regardless of whether the person being represented by a DD is dead or alive).

This raises a fundamental question that Iglesias and colleagues do not fully address, and which is not relevant when only considering biological life extension: how can we ever ensure who has ultimate control over a digital doppelgänger’s evolution *over time*? While initial parameters might be set by the individual before death, corporations hosting the DD would likely need to update and modify these systems as technology advances. This tension between preserving authenticity and maintaining functionality poses a significant challenge to Iglesias et al.’s argument about D-relations extending aspects of personhood beyond death.

## REASSESSING D-RELATIONS IN LIGHT OF CORPORATE CONTROL

Iglesias and colleagues present a thought experiment where, faced with an imminent nuclear strike, an individual must choose between death without any continuation, or the creation of a digital doppelgänger through a “Duplicator” machine. They suggest that while this D-relation replica may lack consciousness, it might still be preferable to complete obliteration, as it could fulfill certain aims of personhood extension like maintaining relationships and preserving influence. They also specify that the Duplicator is technologically and ethically advanced enough to keep private data secure, ensuring that intimate information is only used under appropriate conditions.

It is easy to stipulate these safety features, but they distort the intuitions this thought experiment is pumping, as they are less convincing when we take into account the very long-term. This is because the Duplicator allows for the possibility of exploitation that could persist *indefinitely—*and, indeed, could commence only in the distant future. Unlike biological death, which limits the ease with which one’s identity can be used, DD creation creates limitless opportunities for manipulation, at least if we account for the very long-term. While medical companies may profit from extending lifespans, they cannot fundamentally alter a living person’s beliefs, values, or relationships in the same way a digital doppelgänger could be manipulated.

The *permanence* of this risk matters. A DD that gradually shifts away from authentic representation doesn’t just fail to preserve personhood—it actively shapes how someone is remembered. Family members who maintain relationships with DDs might find their memories slowly reshaped by interactions that increasingly serve corporate or other non-familial interests. What begins as a way to maintain connections or complete projects will always be vulnerable to evolving into something that distorts rather than preserves relationships and legacy.

These implications also extend into considerations of posthumous welfare, at least for those who accept that what someone is associated with and how they are remembered can affect their posthumous well-being—perhaps through a preference satisfaction frame­work. On such accounts of well-being, the potential harm becomes both real and, troublingly, boundless. A DD that continues to exist after death can be corrupted or exploited indefinitely, and as long as the digital self persists, there’s no true endpoint to the potential harm inflicted.

This introduces the prospect of potentially unbounded negative welfare. Unlike biological death, where an individual’s suffering or exploitation ends when their existence does, DDs theoretically exist forever. A corrupted DD might endure decades, even centuries, of misuse—subject to evolving technological capabilities, shifting ownership, and changing societal attitudes.

## CONCLUSION

Iglesias and colleagues suggest that D-relations offer a kind of consolation—a second or third best solution for those who wish to sustain some form of continuity when biological life is no longer possible. Yet, the risks of indefinite exploitation—raised by corporate ownership of the underlying infrastructure but not limited to it—fundamentally challenge this intuition. In choosing to create a DD, we do not simply choose continuity over oblivion; we also choose a *type* of vulnerability that might be worse than not being able to maintain certain aspects of relationships or completing valuable projects.

In the nuclear attack scenario, then, the wager must be carefully reconsidered: it is not simply between nothingness and the benefits of digital continuation. Instead, the choice involves weighing complete obliteration against the possibility of fulfilling important projects or maintaining relationships, all while accepting the timeless potential for manipulation in the very long-term (something that their stipulation of safety feature at the time of use does not quite satisfy). While they make a compelling case for D-relations as better than nothing, the introduction of indefinite interference potential adds a significant dimension of risk that their analysis does not fully address. The very immortality that makes DDs attractive as a form of persistence also makes them permanently vulnerable to exploitation.

## Footnotes

1 As Iglesias et al. (2025) note, these include hereafter.ai, but also DeepBrain AI. See Jeong and Kim (2024).

2 For a discussion on “griefbots”, see: Hollanek and Nowaczyk-Basińska (2024).

3 At least when it comes to large language models. See Liu (2024).
